# Piloting the Effectiveness of a Text Messaging and Fitness Tracking Intervention within Older Black Women

**DOI:** 10.1093/geroni/igab046.2889

**Published:** 2021-12-17

**Authors:** Olivio Clay, Veronica Mixon, William Opoku-Agyeman, Meneka Johnson Nicholson, Pamela Bowen

**Affiliations:** 1 University of Alabama at Birmingham, Birmingham, Alabama, United States; 2 UAB, Birmingham, Alabama, United States; 3 Healthcare Management, University of North Carolina at Wilmington, North Carolina, United States; 4 Rural Health Medical Program, Inc., Selma, Alabama, United States; 5 The University of Alabama at Birmingham, Birmingham, Alabama, United States

## Abstract

Physical activity (PA) can help lower risk of obesity and type 2 diabetes, reduce anxiety, and reduce risk of Alzheimer’s and other related dementias. Despite these benefits, older, obese Black women are not meeting CDC recommended PA guidelines at disproportionate rates. This study aims to identify whether a targeted intervention, Texting Older Sisters to Step (T.O.S.S.), can improve health-related outcomes within older Black women. A sample of 24 Black women (12 per group) age 60 and older who had a BMI > 30 were recruited. The treatment group received text messages previously validated to promote physical activity every day for 12-weeks and were placed in Fitbit communities. The control group received a general health or nutrition-related text message every Sunday. Participants ranged from 60 to 70 years of age with a mean of 64 and 90% had at least some college education. Overall, there was a significant reduction of 1.53 inches in waist circumference, p < .01. When the groups were compared, the treatment group showed a 2.16 inch reduction compared to a 0.91 inch reduction in the control group (Cohen’s d=0.54, a medium effect size). Similarly, the treatment group lost 2.50 pounds on average compared to 1.33 in the control group (d=0.23). When the groups were compared on HgA1c, the treatment group was stable with a reduction of 0.01 unit whereas the control group reduction was 0.15 unit (d=0.23). Findings provide initial support for the T.O.S.S. intervention and suggest a modification of including nutrition information among the intervention messages.

